# Treatment of Goitre by Electricity

**Published:** 1890-03-29

**Authors:** 


### TREATMENT OF GOITRE BY ELECTRICITY.

Dr. Marano reports the complete cure of a goitre of considerable size and of three years' duration, which seriously interfered with deglution. Internal remedies and external applications having entirely failed to check its growth, he tried the effects of electricity, in the form of strong faradic currents externally and electrolysis alternately. For the latter purpose he used five to eight Leclanché's cells, a sponge held between the patient's hands for the positive pole, and connected with the negative pole, a glover's needle, which he inserted into the tumour. On two occasions he connected both poles with needles plunged into the gland. One or two electrolytic and one to three faradic séances were given weekly, the former lasting for five minutes, and the latter ten to fifteen. Improvement appeared after the first séance in the subjective symptoms, a marked diminution of the tumour was visible by the sixth; and though after the first month the progress was less rapid than previously, the swelling ultimately disappeared completely.

